# Melanosis coli in a peritoneal dialysis patient: a case report

**DOI:** 10.1186/s13256-021-02895-2

**Published:** 2021-07-30

**Authors:** Nor Fadhlina Zakaria, Nurul Izah Ahmad, Elmina Mokhtar, Wan Zul Haikal Hafiz Wan Zukiman, Anim Md Shah

**Affiliations:** 1 Faculty of Medicine and Health Sciences, University Putra Malaysia, 43400 Serdang, Selangor Malaysia; 2 Nephrology Department, Serdang Hospital, 43400 Serdang, Selangor Malaysia

**Keywords:** Melanosis coli, Peritoneal dialysis, Diarrhea

## Abstract

**Background:**

Patients who undergo peritoneal dialysis (PD) are at risk of gut bacteria translocation leading to peritonitis when there is chronic diarrhea. Chronic diarrhea is defined as any course of diarrhea that lasts at least 4 weeks, which can be continuous or intermittent. Chronic diarrhea of any duration may cause dehydration, electrolyte imbalance, and life-threatening hypovolemic shock. In PD patients, excessive ultrafiltration from the exchanges, combined with severe gastrointestinal loss, may cause hypovolemic shock, electrolyte imbalance, and metabolic acidosis. There are multiple causes of chronic diarrhea in PD patients including infective causes, mitotic lesions, and rarely the regular and excessive use of laxatives, which is a diagnosis of exclusion.

**Case presentation:**

We report a case of Melanau lady with chronic diarrhea secondary to laxative usage in a patient being treated with automated peritoneal dialysis (APD). The patient went into hypovolemic shock, but luckily did not contract peritonitis. A colonoscopy revealed brown to black discoloration of the colon, a feature suggestive of melanosis coli. A biopsy of the intestine further confirmed the diagnosis by histopathological examination. Withdrawal of laxatives and the introduction of probiotics improved the symptoms tremendously.

**Conclusions:**

The chronic use of laxatives in PD patients can potentially lead to a devastating problem; thus, the management team must monitor treatment commencement appropriately.

## Background

Patients undergoing peritoneal dialysis (PD) may have hypomotility disorders due to frequent uremia [1]. They are also on drugs that contribute to constipation, such as calcium-based phosphate binders and iron supplements. Constipation is a risk factor for peritonitis due to intestinal bacterial translocations [2]. It has also been reported as a cause of mechanical failure of the catheter. Generally, patients on PD need at least one bowel movement daily to help maintain the PD catheter position and avoid poor outflow caused by the "impacted" tube. During the PD training, regular bowel movements and constipation avoidance are emphasized to all patients; hence, chronic laxative use in PD patients is not uncommon. However, laxative usage can cause chronic diarrhea, which can be debilitating, especially in the elderly. We report a case of melanosis coli due to regular and excessive laxative usage in a patient undergoing automated peritoneal dialysis (APD).

## Case presentation

A 65-year-old Melanau (native Malaysian) lady was diagnosed with end-stage kidney disease (ESKD) secondary to diabetes in March 2020 during a routine follow-up, but was asymptomatic. She was a widower, lost her husband 3 years before the ESKD diagnosis, and had three supportive daughters who were always there during her illness. She never smoked or consumed alcohol. There was no surgical history, and she was compliant with her insulin, low-dose antihypertensive medications, phosphate binders, and vitamins all along. She opted for PD treatment, mainly APD, due to her lifestyle choices. However, before the elective PD catheter insertion, she presented with uremic symptoms in July 2020. We inserted a Tenckhoff catheter for her via peritoneoscopy, and a home-based APD training was started 2 weeks after the insertion. She was given lactulose 15 mL three times per day and bisacodyl 10 mg at night after the procedure to ensure bowel movement. She was well up to the time of the APD training. She reported having two to three bowel movements in a day, which she attributed to laxatives to prevent constipation and the dialysate fill in the abdomen. She had good ultrafiltration (UF), resulting in an asymptomatic episode of hypotension during the training. Her effluent was clear, and she had no abdominal pain during the bouts of diarrhea. Subsequently, with reasonable reassurance, she was advised to withhold her antihypertensive medications and stop the laxative usage. Her diarrhea improved, and her blood pressure returned to normal.

She managed to complete the APD training successfully. Her regime was 10 L of 1.5% dextrose dialysate every day, and her UF was to 500 mL/day with residual urine output of 300 mL/day. She reported having a bowel movement only once a day; thus, she was subsequently advised to retake the osmotic laxative (lactulose 15 mL three times daily) to maintain bowel output at least once to twice per day. She complied with all the prescriptions and was scheduled for a clinic follow-up about a month later.

Upon review at follow-up, she complained of diarrhea containing excessive liquid for 4 weeks. The frequency was 2–3 times per day initially but increased to more than 10 times per day a week before the follow-up. There was no cloudy effluent, vomiting, or fever, but she complained of colicky abdominal pain, nausea, and appetite loss associated with diarrhea. In the past month, she had lost 10 kg, but she thought it was a standard expectation for a new dialysis patient. Upon further questioning, she revealed a family history of endometrial carcinoma but no family history or contact with tuberculosis patients. On examination, she appeared dehydrated; however, she was alert and not in distress. Her blood pressure on admission was 108/52 mmHg and pulse rate was 85 beats per minute, regular rhythm. She was afebrile and not in respiratory distress. Abdominal examination revealed a soft, non-tender abdomen, and there was no organomegaly. Respiratory, neurological, and other examinations were unremarkable. Greenish fluffy pieces with ragged edges and mushy stool (Bristol stool 6) [3] were seen in stool inspection. PD fluid was clear, with zero cell counts and negative culture. No pathogen was isolated in the stool in blood culture and sensitivity tests. Unfortunately, no stool osmolality or stool osmolar gap test was done for this patient, as it was not available in our center. Her full blood counts and electrolytes (mainly potassium and sodium) were surprisingly normal despite frequent diarrhea and dehydration. Only her C-reactive protein was mildly elevated to 34 mg/L (0–5 mg/L), indicating ongoing inflammation. However, her tumor markers were all negative, and thyroid function test was normal. Table 1 summarizes her initial blood investigations on admission.

**Table 1:** Investigations results upon admission

Investigations	Results		Reference values
Full blood counts	Hemoglobin Total white cell count Platelet Mean corpuscular volume Mean corpuscular hemoglobin	9.1 10.8×10^9^ 391 95 30	11.6–15.1 g/dL 4.0–11.4 × 109/L 150–400 × 109/L 80.6–95.5 fL 26.9–32.3 pg
Renal profile and electrolytes	Urea Sodium Potassium Creatinine Calcium Phosphate	10.0 135 4.7 1031 1.7 1.46	2.5–7.1 mmol/L 135–145 mmol/L 3.5–5.0 mmol/L 44–97 μmol/L 2.2–2.6 mmol/L 0.84–1.45 mmol/L
Liver function test	Albumin Total bilirubin Alkaline phosphatase Aspartate transaminase Alanine aminotransferase	24 9.8 80 49 10	35–50 g/L 4–20 U/L 45–145 IU/L 8–35 IU/L 10–45 IU/L
Blood, stool, urine, and peritoneal dialysis fluid culture and sensitivity	Negative		

**Fig. 1:**
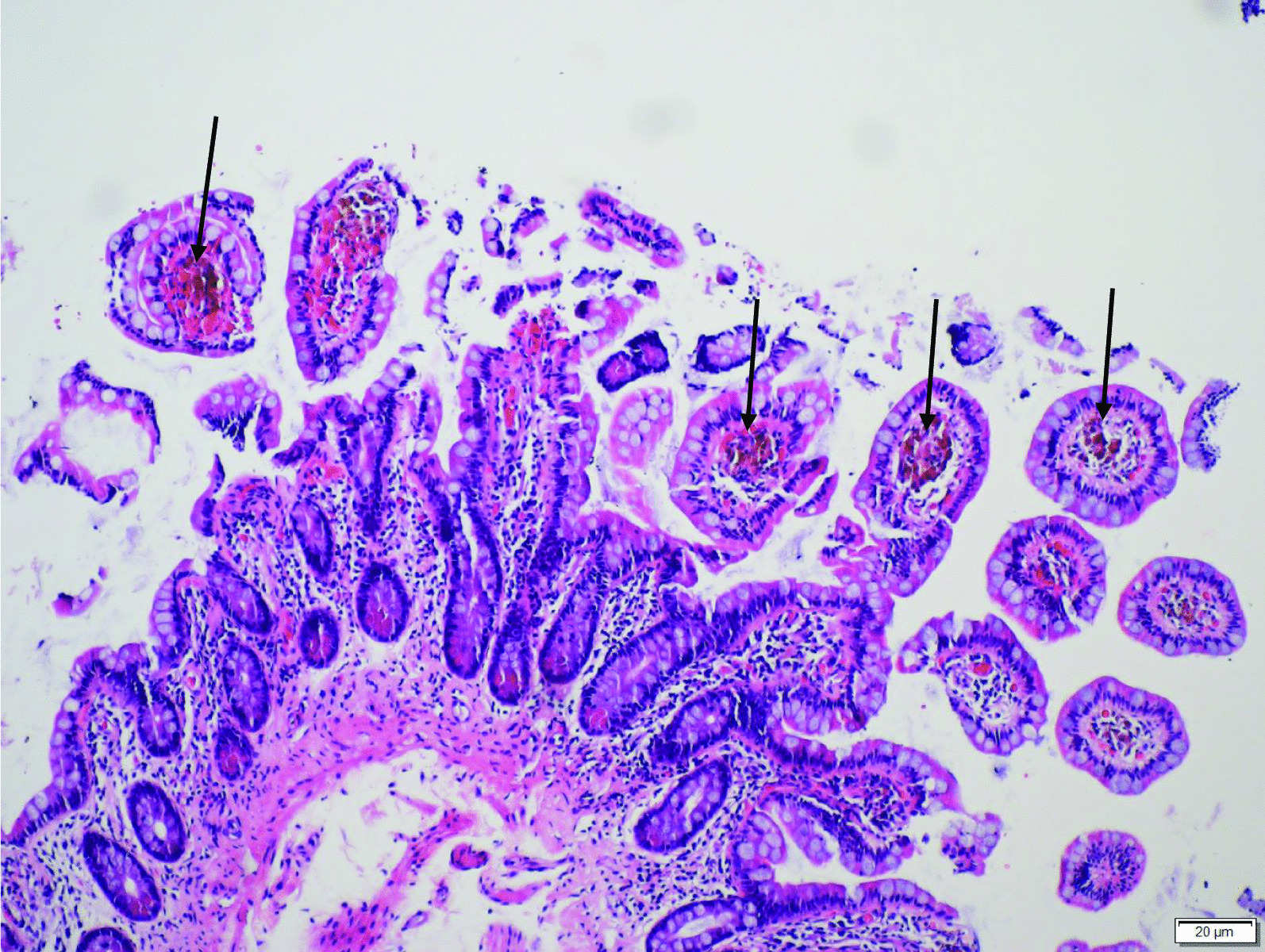
Macrophages containing brown pigments (arrows) are evident in the lamina propria of terminal ileal biopsy, particularly at the tips of the villi (hematoxylin and eosin stain, ×100)

**Fig. 2:**
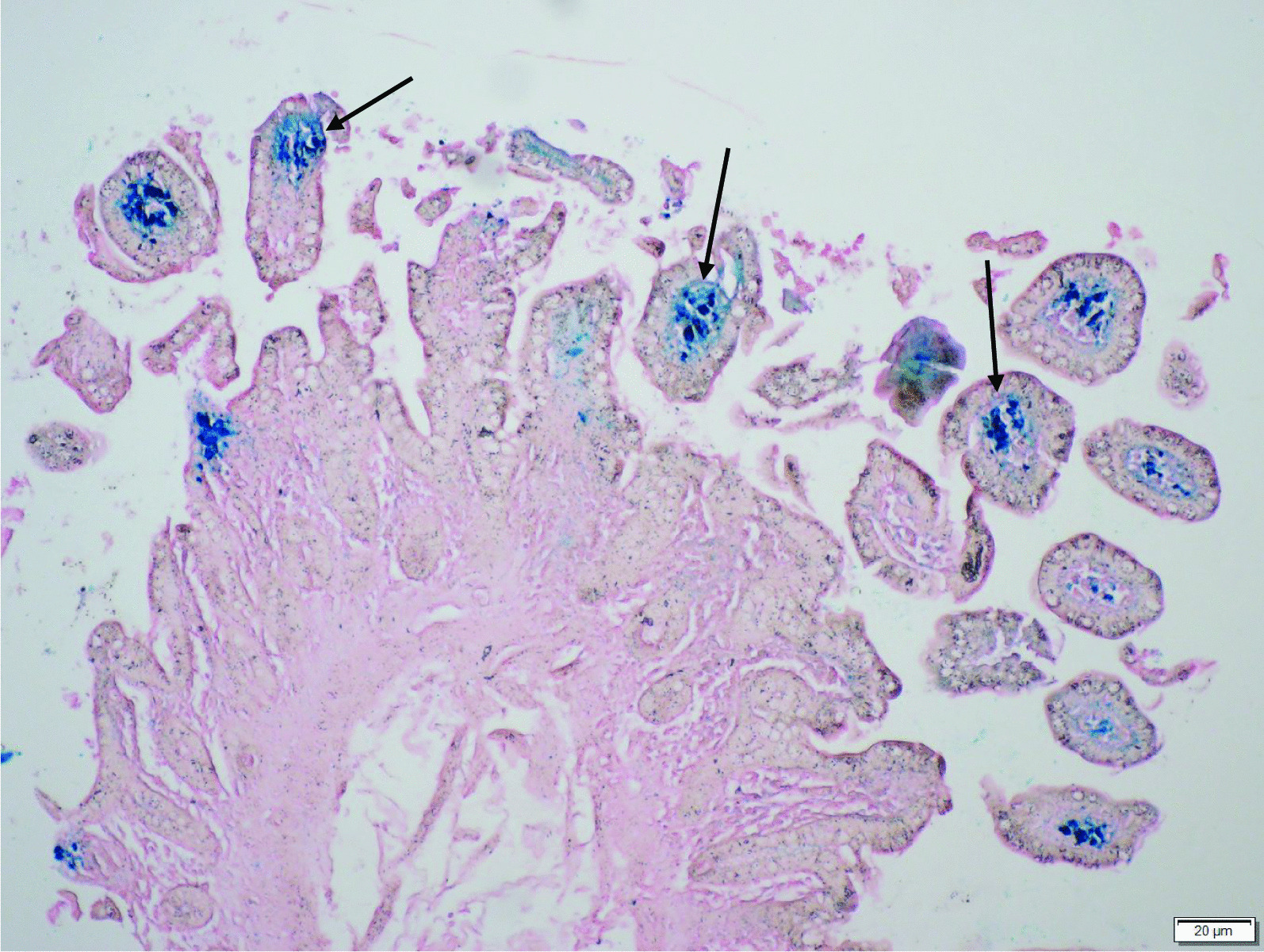
Perls Prussian blue stain (arrows) is positive, indicating that the pigments are hemosiderin (×100)

We resuscitated her with saline and withheld all her laxatives immediately. An abdominal radiograph and abdominal ultrasound did not reveal any abnormality. We proceeded to a colonoscopy examination that revealed brown to black discoloration over the cecum, ascending, transverse, descending, and sigmoid colon and rectum, clinically suggestive of melanosis coli. Unfortunately, no pictures of the colon were taken during the procedure. Histopathological analyses of the tissues further confirmed the diagnosis, as numerous macrophages containing granular, coarse, brown lipofuscin-like pigments (Perls stain negative) were found within the lamina propria (Figs. 1 and 2). Her stool culture came back as no growth, excluding any infective origin of her diarrhea.

The laxative prescription was stopped completely, and the dietitian gave her partially hydrolyzed guar gum (PHGG; precursor for probiotics), two scoops a day (about 15 g/day) orally, which improved her symptoms significantly. The PHGG was prescribed for 7 days as her diarrhea resolved substantially. She was discharged well after the restoration of volume and resumed her usual dialysis. On her follow-up 6 months later, she had been relatively well and had no more diarrhea complaints. She felt better, and her recorded blood pressures were normal. Nevertheless, she still continues her APD.

## Discussion

Our patient, who had just started her ESKD treatment, developed a life-threatening condition after severe diarrhea attributed to the chronic use of laxatives. Once peritonitis had been ruled out, we embarked on investigating chronic diarrhea, which intestinal biopsy revealed to be melanosis coli. For most PD patients with diarrhea, the management team must always rule out infection as the primary cause. However, given no other signs to suggest an infective cause, other differentials of chronic diarrhea must be considered. They should be addressed appropriately, especially in the PD population, in which laxatives are commonly used for bowel care.

Melanosis coli refers to a benign condition in which the lining of the colon and rectum, usually pink in color, turns to black or brown [4]. Variable incidence of melanosis coli has been reported in many previous studies and is common in women and the elderly [5–7]. Its incidence among dialysis patients is not well established; however, Zhang *et al.* reported a rate of 19.2% among ESKD patients awaiting kidney transplantation in China [8]. The frequent use of rhubarb-containing traditional Chinese herbs (anthraquinone) was thought to be a contributing factor. A well-known cause of melanosis coli is the use of laxatives, especially anthraquinone [9, 10]. To date, no study has reported on the occurrence of melanosis coli in a PD population. Our patient had been put on an osmotic laxative (lactulose) and stimulant laxative (bisacodyl) as routine medications in her bowel care regime.

The Bristol Stool Chart can be used to ascertain the severity of bowel movements. This chart classifies the stool sample (shape and consistency ) and assigns it to type 1–7, depending on its characteristics [3]. For example, type 3 and 4 stools that are well formed and easy to pass are considered standard. Conversely, stool that is hard and difficult to pass (types 1 and 2) indicates constipation, and type 6 and 7 stools indicate diarrhea.

Type 1: Separate hard lumps (hard to pass)

Type 2: Lumpy, sausage-shaped

Type 3: Sausage-shaped with cracks on the surface

Type 4: Sausage-shaped or snake-like; smooth and soft

Type 5: Soft blobs with clear-cut edges (easy to pass)

Type 6: Fluffy pieces with ragged edges; mushy

Type 7: Entirely liquid, watery, no solid pieces

Our patient presented with chronic diarrhea associated with significant weight loss and increased bowel movement for a month after her PD training. As she presented with dehydration signs during follow-up, she was admitted for further workup of her chronic diarrhea symptoms. Once we excluded the commonest cause of diarrhea in the PD population, which is peritonitis, we conducted further investigation. A colonoscopy revealed a brownish discoloration in a repetitive pattern seen in the mucosa on endoscopy. Some lesions of the colonic mucosa may be brownish or blackish. The pigment can be observed more intensely in the cecum and ascending colon than in the distal column [6, 11]. This pigment probably originates from organelles of epithelial cells or macrophages, which are damaged by treatment. In our case, melanosis coli was detected throughout the colon. A tissue biopsy will confirm the diagnosis with histological findings of pigment-laden macrophages in the lamina propria. Three grades of melanosis coli are described depending on the extent of pigmentation, which is due to the phagocytosis of apoptotic cells and accumulation of pigmented phagosomes in the macrophages [12].

Evaluating patients with chronic diarrhea, especially melanosis coli, requires several clues to support the diagnosis. A proper history of medications, past medical history, and comprehensive examination must be made accordingly. Factitious diarrhea is a differential diagnosis that one must rule out, especially when the clinical suspicion is high. Laboratory studies should include stool osmolality, electrolytes, osmotic gap, and a laxative screen. Laboratory investigations including a complete blood count and differential, C-reactive protein, sedimentation rate, serum electrolytes, total protein, and albumin must be available to assess the volume and nutritional status and detect electrolyte abnormalities. Colonoscopy is part of the evaluation for chronic diarrhea.

The initial management of chronic diarrhea consists of correction of electrolyte abnormalities, dehydration, and malnutrition. However, the treatment of melanosis coli involves discontinuation of laxative usage. The illness can develop within 4 months of laxative initiation, especially those containing anthraquinone, and can disappear in the same amount of time if laxative use is discontinued. Some studies have shown that the symptoms might recede within weeks to months [10, 12]. In this case, the introduction of probiotics in PHGG helped improve symptoms, but further studies can be done to determine the correlations clinically. PHGG is a soluble dietary fiber, completely dissolves in water, does not form a gel, and demonstrates prebiotic properties [13]. As far as the risk of developing carcinoma is concerned, a survey done by Biernacka-Wawrzonek and colleagues showed that the cause–effect relationship between melanosis coli and colon cancer remains uncertain [4]. Some reports indicate that the risk of colorectal cancer increases with non-fiber laxative use and decreases with fiber laxative use [14].

## Conclusion

The presence of melanosis coli poses a minor threat to PD patients. It is a benign disease, which does not carry any high risk of complications for the patients. Nevertheless, if the severity of diarrhea, coupled with good UF from the PD regime, causes life-threatening dehydration, one must take this as a severe threat to the patient. Balancing good bowel movements and inducing diarrhea using laxatives in PD patients requires a delicate approach by the management team, including a nephrologist, PD nurses, and dieticians.

## Data Availability

Not applicable.

## Abbreviations

APD: Automated peritoneal dialysis
ESKD: End-stage kidney disease
PD: Peritoneal dialysis
UF: Ultrafiltration
PHGG: Partially hydrolyzed guar gum

## References

[1] Hirako M, Kamiya T, Misu N, Kobayashi Y, Adachi H, Shikano M, Matsuhisa E, Kimura G. Impaired gastric motility, and its gastrointestinal relationship symptoms in patients with chronic renal failure. J Gastroenterol. 2005;40(12):1116–22. 10.1007/s00535-005-1709-6 16378175

[2] Leung L, Riutta T, Kotecha J, *et al*. Chronic constipation: an evidence-based review. J Am Board Fam Med. 2011;24(4):436–51. 10.3122/jabfm.2011.04.100272 21737769

[3] Chumpitazi BP, Self MM, Czyzewski DI, Cejka S, Swank PR, Shulman RJ. Bristol Stool Form Scale reliability and agreement decreases when determining Rome III stool form designations. Neurogastroenterol Motil. 2016;28(3):443–8. 10.1111/nmo.12738 PMC4760857 26690980

[4] Biernacka-Wawrzonek D, Stepka M, Tomaszewska A, Ehrmann-Josko A, Chojnowska N, Zemlak M, *et al*. Melanosis coli in patients with colon cancer. Prz Gastroenterol. 2017;12:22–7. 10.5114/pg.2016.64844 PMC5360664 28337232

[5] Wang S, Wang Z, Peng L, Zhang X, Li J, Yang Y, Hu B, *et al*. Gender, age, and concomitant diseases of melanosis coli in China: a multicenter study of 6,090 cases. PeerJ. 2018;6:e4483. 10.7717/peerj.4483 PMC5845562 29568709

[6] Nesheiwat Z, Al NY. Melanosis coli. Treasure Island: StatPearls Publishing; 2019.

[7] Siegers CP, von Hertzberg-Lottin E, Otte M, Schneider B. Anthranoid laxative abuse-a risk for colorectal cancer? Gut. 1993;34:1099–101. 10.1136/gut.34.8.1099 PMC1374362 8174962

[8] Zhang WX, Zhou W, Li Y, Wang Y, Huang W High detection rate and risk factors of melanosis coli in end-stage renal disease patients awaiting kidney transplantation: a single-center retrospective study in China. Res Square. 2020. 10.21203/rs.3.rs-65504/v2.

[9] Bockus HL, Willard JH, Bank J. Melanosis coli: the etiologic significance of the anthracene laxatives: a report of forty-one cases. JAMA. 1933;101(1):1–6.

[10] Hung CY, Shyung LR, Chen MJ. Pigmentation sparing on melanosis coli. Gastroenterology. 2012;142(3):e10–1. 10.1053/j.gastro.2011.07.032 22285363

[11] Van Gorkom BA, Karrenbeld A, van der Sluis T, Zwart N, de Vries EG, Kleibeuker JH. Apoptosis induction by sennoside laxatives in man; escape from a protective mechanism during chronic sennoside use? J Pathol. 2001;194(4):493–9. 10.1002/path.914 11523059

[12] Zhang L, Gao F. New progress of melanosis coli. Chin J Gastroenterol Hepatol. 2015;24(3):257–9.

[13] Yasukawa Z, Inoue R, Ozeki M, Okubo T, Takagi T, Honda A, Naito Y. Effect of repeated consumption of partially hydrolyzed guar gum on fecal characteristics and gut microbiota: a randomized, double-blind, placebo-controlled, and parallel-group clinical trial. Nutrients. 2019;11:2170. 10.3390/nu11092170. PMC6769658 31509971

[14] Citronberg J, Kantor ED, Potter JD, White E. A prospective study of the effect of bowel movement frequency, constipation, and laxative use on colorectal cancer risk. Am J Gastroenterol. 2014;109(10):1640–9. 10.1038/ajg.2014.233 PMC4734897 25223576

